# Refractory Lactic Acidosis in Small Cell Carcinoma of the Lung

**DOI:** 10.1155/2017/6148350

**Published:** 2017-05-23

**Authors:** Daniel J. Oh, Ellen Dinerman, Andrew H. Matthews, Abraham W. Aron, Katherine M. Berg

**Affiliations:** ^1^Department of Medicine, Beth Israel Deaconess Medical Center, Harvard Medical School, 330 Brookline Ave, Boston, MA 02215, USA; ^2^Department of Medicine, Division of Pulmonary, Critical Care and Sleep Medicine, Beth Israel Deaconess Medical Center, Harvard Medical School, 330 Brookline Ave, Boston, MA 02215, USA

## Abstract

**Background:**

Elevated lactate levels in critically ill patients are most often thought to be indicative of relative tissue hypoxia or type A lactic acidosis. Shock, severe anemia, and thromboembolic events can all cause elevated lactate due to tissue hypoperfusion, as well as the mitochondrial dysfunction thought to occur in sepsis and other critically ill states. Malignancy can also lead to elevation in lactate, a phenomenon described as type B lactic acidosis, which is much less commonly encountered in the critically ill.

**Case Presentation:**

We present the case of a 73-year-old Caucasian woman with type 2 diabetes and hypertension who presented with abdominal pain, nausea, vomiting, nonbloody diarrhea, and weight loss over five weeks and was found to have unexplained refractory lactic acidosis despite fluids and antibiotics. She was later diagnosed with small cell carcinoma of the lung.

**Conclusions:**

In this case report, we describe a critically ill patient whose elevated lactate was incorrectly attributed to her acute illness, when in truth it was an indicator of an underlying, as yet undiagnosed, malignancy. We believe this case is instructive to the critical care clinician as a reminder of the importance of considering malignancy on the differential diagnosis of a patient presenting with elevated lactate out of proportion to their critical illness.

## 1. Case Vignette

The patient is a 73-year-old Caucasian woman with type 2 diabetes and hypertension who presented to a local community hospital with abdominal pain, nausea, vomiting, nonbloody diarrhea, and weight loss over five weeks. She was afebrile, blood pressure was 148/77, and she was tachycardic to 109 beats per minute, and exam was notable for mild diffuse tenderness in her abdomen and an enlarged liver on palpation. Her laboratory studies revealed a WBC of 12,600 cells/*μ*L, bicarbonate of 11 mEq/L, BUN of 29 mg/dL, and creatinine of 1.2 mg/dL with an anion gap of 30. Troponins were undetectable and lactate was elevated at 4.9 mmol/L. Mild transaminitis was present with ALT of 32 U/L and AST of 50 U/L; alkaline phosphatase was elevated at 231 U/L. However, normal values included albumin of 4.3 g/dL and INR of 1.0.

A chest X-ray was unrevealing. An abdominal CT showed a well-defined lesion in the superior right hepatic lobe consistent with a hepatic cyst and a mildly enlarged and heterogeneous left hepatic lobe concerning for possible hepatocellular carcinoma. The differential diagnosis included diabetic ketoacidosis, metformin-related injury, bowel ischemia, liver cirrhosis, and malignancy. Workup including viral hepatitis, CA-19, ceruloplasmin, anti-mitochondrial antibody, tissue transglutaminase IgA, alpha-fetoprotein, and alpha-1- antitrypsin was unremarkable.

She was admitted for further workup, and her course was complicated by hematemesis. An esophagogastroduodenoscopy (EGD) showed stable ulcers with no need for cauterization. After 2 weeks in the hospital with no etiology for her symptoms being discovered and with persistent abdominal pain and nausea, she left the hospital against medical advice due to frustration. Lactate at discharge was elevated at 14.5 mmol/L with anion gap of 31.

The next morning, the patient returned to the outside hospital after being found in her room in a pool of blood with altered mental status. She was afebrile, BP was 101/47, and she was tachycardic to 115 beats per minute. Her hematocrit dropped to 21% and lactic acid was 25 mmol/L. Her arterial blood gas pH was 6.8, PaCO_2_ was 21 mmHg, and PaO_2_ was 43 mmHg. She was given bicarbonate, started on a Protonix drip, and transfused with 2 units of red blood cells with appropriate hematocrit increase to 25%. She was intubated for airway protection. Plans were made to transfer her to a tertiary care center given her declining condition and unclear lactate etiology and for repeat EGD.

**Figure 1:**
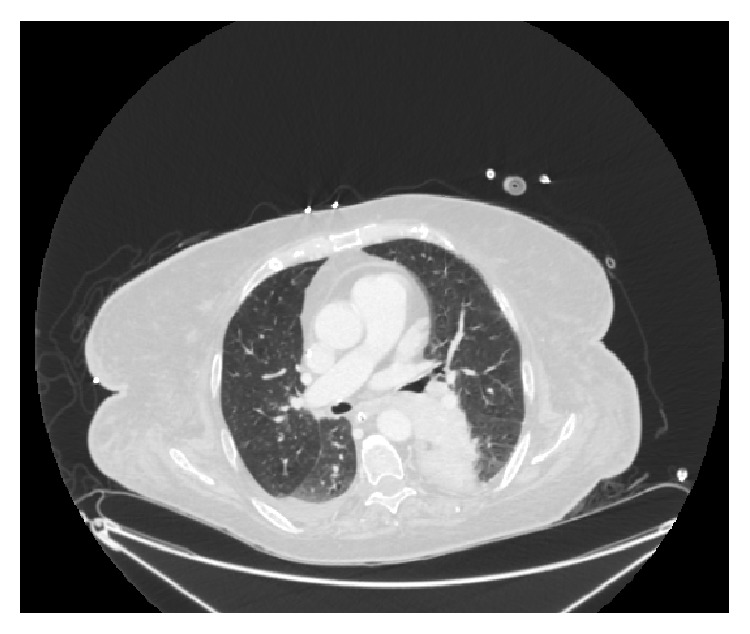
Computed tomography of the chest showing a large mediastinal obstructive mass in the left chest.

**Figure 2:**
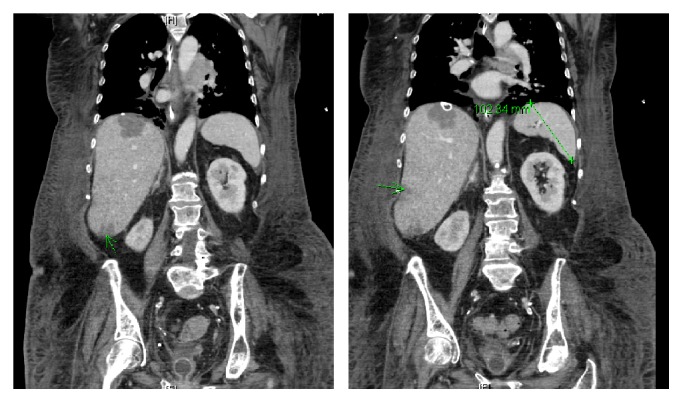
Cross sections of computed tomography of the torso showing scattered, ill-defined, hypoattenuating lesions (green arrows) in the right hepatic lobes of an enlarged liver. Spleen is found to be normal in size, measuring 10.2 cm in length.

On transfer to our hospital, the patient was intubated and sedated and was requiring vasopressors. Labs showed a leukocytosis at 22,000 cells/*μ*L and lactate was persistently elevated but improved at 8.3 mmol/L. The improvement in heart rate and lactate was initially thought to be from fluids and vasopressors. Her lactate decreased to a low of 4.6 mmol/L, but, in spite of the subsequent resolution of her shock, the lactate rose again to 9.4 mmol/L. EGD showed bleeding ulcers requiring epinephrine injection, and there was no recurrence of hematemesis after that procedure. Given her leukocytosis and initial hypotension, she was also given vancomycin, piperacillin-tazobactam, and metronidazole. To evaluate for a source of presumed sepsis, a CT scan of the chest was done. This revealed a large mass in the left posterior mediastinum with associated consolidation concerning for postobstructive pneumonia (Figure 1). Bronchoscopy revealed purulent secretions and biopsy of the mass demonstrated small cell carcinoma. The CT abdomen revealed a liver with a nodular contour and hypertrophy of segments of the hepatic lobes, suggestive of cirrhosis. The same questionable hypoattenuating lesion, initially thought to be a hepatic cyst from the outside hospital, was seen. Other scattered, ill-defined lesions were also observed and were interpreted as suspicious for metastatic disease (Figure 2).

Chemotherapy was recommended but the patient and family declined aggressive care and she was transitioned to comfort measures only. On the day of her passing, she was no longer requiring vasopressors. Her WBC was elevated at 28,600 cells/*μ*L, ALT was 132 U/L, AST was 162 U/L, alkaline phosphatase was 992 U/L, albumin was 1.8 g/dL, INR was 1.4, and lactate remained elevated at 16.5 mmol/L.

## 2. Discussion

Elevated lactate has been associated with increased mortality in patients with hematological malignancies. However, its presence in solid tumors such as small cell carcinoma of the lung has been reported rarely [1–5]. Even fewer cases have been described in the setting of critical care where comorbid conditions such as shock, hypoxemia, and end-organ failure can confound the etiology of the elevation in lactate.

This case report illustrates that type B lactic acidosis must be considered, especially in the case when there is elevated lactate without hypotension as was the case on the patient's initial admission. Her other markers of ischemic damage, including troponins and creatinine, were normal. Her lactic acidosis worsened in the setting of subsequent septic and hypovolemic shock but, even after successful resuscitation, the lactate was persistently elevated. Solid tumors leading to type B lactic acidosis usually result from metastases to the liver given liver involvement in lactate metabolism [2]. While she had suspicious imaging findings in her liver, the patient refused a definitive biopsy.

The pathophysiology of type B lactic acidosis from malignancy is believed to be multifactorial including altered lactate metabolism from liver and kidney dysfunction and lactate production by cancer cells because of tumor cell overproduction of growth factors promoting glycolysis even under aerobic conditions, termed the Warburg effect [6]. This may be due to adaptations in malignant cells to low-oxygen environments within tumors or the relative efficiency of glycolysis as it provides most of the metabolic products for cells to proliferate. With increased end-product pyruvate, there is also increased lactate, which can lead to type B lactic acidosis. In our patient, while sepsis physiology likely contributed to her elevated lactate levels initially, her lactate continued to rise even when she was no longer requiring any vasopressors. In addition, there is evidence of markedly worsening hepatic dysfunction, indicated by the change in her liver function tests, albumin, and INR from initial presentation to her expiration. Her hepatic laboratory abnormalities in addition to the ill-defined imaging findings in the liver may indicate possible metastatic involvement of the liver, although the family declined a formal biopsy. These findings argue for type B lactic acidosis from her malignancy and hepatic dysfunction contributing a significant role in her refractory lactic acidosis.

High lactate level in type B lactic acidosis has been documented as being associated with significantly shorter overall survival [7]. Similarly, lactate resolution has been noted after the initiation of chemotherapy. With recurrence of solid tumors, lactic acidosis has been observed to recur [1, 3]. However, other studies note that elevated lactate levels in solid tumors are not predictive of prognosis or recurrence. In the case of our patient, given her desire to forgo chemotherapy, these variables could not be assessed and her lactate continued to rise until her expiration.
